# Case Report of Dual-Site Neurostimulation and Chronic Recording of Cortico-Striatal Circuitry in a Patient With Treatment Refractory Obsessive Compulsive Disorder

**DOI:** 10.3389/fnhum.2020.569973

**Published:** 2020-10-23

**Authors:** Sarah T. Olsen, Ishita Basu, Mustafa Taha Bilge, Anish Kanabar, Matthew J. Boggess, Alexander P. Rockhill, Aishwarya K. Gosai, Emily Hahn, Noam Peled, Michaela Ennis, Ilana Shiff, Katherine Fairbank-Haynes, Joshua D. Salvi, Cristina Cusin, Thilo Deckersbach, Ziv Williams, Justin T. Baker, Darin D. Dougherty, Alik S. Widge

**Affiliations:** ^1^Department of Psychiatry, Medical School, University of Minnesota Twin Cities, Minneapolis, MN, United States; ^2^Athinoula A. Martinos Center for Biomedical Imaging, Massachusetts General Hospital, Charlestown, MA, United States; ^3^Department of Psychiatry, Massachusetts General Hospital and Harvard Medical School, Charlestown, MA, United States; ^4^McLean Institute for Technology in Psychiatry and Harvard Medical School, Belmont, MA, United States; ^5^Department of Neurosurgery, Massachusetts General Hospital, Boston, MA, United States

**Keywords:** neurostimulation, cortico-striatal circuitry, obsessive compulsive disorder, ventral capsule/ventral striatum, supplementary motor area, neural oscillations, synchrony, local field potential

## Abstract

Psychiatric disorders are increasingly understood as dysfunctions of hyper- or hypoconnectivity in distributed brain circuits. A prototypical example is obsessive compulsive disorder (OCD), which has been repeatedly linked to hyper-connectivity of cortico-striatal-thalamo-cortical (CSTC) loops. Deep brain stimulation (DBS) and lesions of CSTC structures have shown promise for treating both OCD and related disorders involving over-expression of automatic/habitual behaviors. Physiologically, we propose that this CSTC hyper-connectivity may be reflected in high synchrony of neural firing between loop structures, which could be measured as coherent oscillations in the local field potential (LFP). Here we report the results from the pilot patient in an Early Feasibility study (https://clinicaltrials.gov/ct2/show/NCT03184454) in which we use the Medtronic Activa PC+ S device to simultaneously record and stimulate in the supplementary motor area (SMA) and ventral capsule/ventral striatum (VC/VS). We hypothesized that frequency-mismatched stimulation should disrupt coherence and reduce compulsive symptoms. The patient reported subjective improvement in OCD symptoms and showed evidence of improved cognitive control with the addition of cortical stimulation, but these changes were not reflected in primary rating scales specific to OCD and depression, or during blinded cortical stimulation. This subjective improvement was correlated with increased SMA and VC/VS coherence in the alpha, beta, and gamma bands, signals which persisted after correcting for stimulation artifacts. We discuss the implications of this research, and propose future directions for research in network modulation in OCD and more broadly across psychiatric disorders.

## Introduction

Obsessive compulsive disorder (OCD) is a chronic and severe psychiatric condition characterized by recurrent and intrusive thoughts, images, or fears which produce marked distress or anxiety (obsessions), and the performance of repetitive mental or physical rituals in response to that anxiety (compulsions). Individuals with OCD experience frequent and significant social impairments (Koran et al., 1996). Roughly 40% of individuals living with OCD report being unable to work (Mancebo et al., 2008). Standard treatments include exposure and response prevention therapy (ERP; e.g., Foa et al., 2005), and pharmacological interventions (e.g., Fineberg and Gale, 2005). Unfortunately, 30–60% of individuals will fail to respond adequately to treatment. Even those who do respond to treatment are often left with some level of residual symptoms (Pallanti et al., 2002; Foa et al., 2005; Dougherty et al., 2018).

For those treatment refractory individuals, neurostimulation, and in particular deep brain stimulation (DBS), is an option. Current neurostimulation therapies arose from the success of psychiatric neurosurgery procedures in which areas of the internal capsule were lesioned, with modern versions of those surgeries having open-label response rates as high as 80% (Brown et al., 2016; Dougherty et al., 2018; Rasmussen et al., 2018; Spatola et al., 2018). Given the irreversible nature of lesion surgeries, neurostimulation was proposed as a reversible option, which has greater customizability than the one-size-fits-all lesion surgeries (Nuttin et al., 1999). Early approaches in the internal capsule evolved into the current ventral capsule/ventral striatum target (VC/VS; Greenberg et al., 2010; Karas et al., 2019; for reports using different names for a similar target see: Luyten et al., 2016; Raymaekers et al., 2017). The VC/VS target is located at a putative junction of the anterior commissure, internal capsule, and striatum (Greenberg et al., 2010). Positive outcomes seen in early open label studies (Greenberg et al., 2010) led the VC/VS target to receive Humanitarian Device Exemption (HDE) approval for OCD in 2009 (approval H050003).

Response and symptom improvement rates with VC/VS DBS are promising, but there is much room for improvement. Reported (Luyten et al., 2016) and non-reported (NCT00640133) randomized controlled trials, as well as open-label trials (Menchón et al., 2019) have shown response rates of around 67% (response is considered a 35% drop in Yale-Brown Obsessive Compulsive Scale – YBOCS). This means that over 30% of individuals did not respond. These studies also found median improvement in YBOCS of 40–60 percent, with a median score of 20 with active stimulation. Critically, a YBOCS of 20 represents a level of symptom severity that often prevents the individual from working (Mancebo et al., 2008). In a qualitative survey of patient perspectives on VC/VS DBS, the majority of patients (86%) cited incomplete or unreliable symptom relief as their primary dissatisfaction with VC/VS DBS (Klein et al., 2016). Therefore, there is a need to advance neurostimulation to produce a more consistent response, and a higher level of effectiveness.

### Improving Neurostimulation for OCD: Potential for Targeted Network Disruption Through Dual-Site Stimulation

Obsessive compulsive disorder is thought to be a network disorder. There is some consensus that dysfunction of the cortico-striatal-thalamo-cortical loops (CSTC loops; e.g., Alexander et al., 1986; Parent and Hazrati, 1995), of which VC/VS (and the striatum, more generally) is a hub (Alexander et al., 1986; Obeso et al., 2008; Krack et al., 2010; Lapidus et al., 2013; Dougherty et al., 2018), is involved in the etiology of OCD (see Dougherty et al., 2018; Robbins et al., 2019 for reviews). Structures outside these loops (e.g., amygdala) also likely play key roles in OCD in at least some patients (Milad and Rauch, 2012; Gürsel et al., 2018; Hazari et al., 2019; Robbins et al., 2019). That said, CSTC loop dysfunction almost certainly plays at least a partial role in OCD. Further, individuals with OCD show deficits in cognitive domains (e.g., cognitive flexibility; Robbins et al., 2012; Shin et al., 2014; Voon et al., 2015; Vaghi et al., 2017) that are thought to involve CSTC loop function (Haber, 2003; Robbins et al., 2012, 2019; Vaghi et al., 2017).

The dominant narrative of CSTC dysfunction in OCD emphasizes CSTC hyper-connectivity (e.g., Dougherty et al., 2018; Calzà et al., 2019). There are many functional neuroimaging studies showing heightened connectivity between regions within CSTC loops (Graybiel and Rauch, 2000; Maia et al., 2008; Milad and Rauch, 2012; Brennan and Rauch, 2017; Dougherty et al., 2018). These have been considered to be further supported by robust results showing striatal hyper-activations in OCD (Robbins et al., 2019), but it is important to recognize that activity and connectivity are entirely separate constructs. A given region may have radically disrupted connectivity without any change in its overall level of activity. In that vein, some studies have linked OCD pathology to hypo- rather than hyper-connectivity within CSTC loop components (Göttlich et al., 2014; Posner et al., 2014; Vaghi et al., 2017). A recent meta-analysis concluded that there is evidence of general aberrant activity in CSTC loops, but that that disconnectivity was not in any specific direction- hypo or hyper (Gürsel et al., 2018). Hyper- versus hypoconnectivity seems to be, in part, a function of which functionally distinct CSTC loop the regions are in Harrison et al. (2009); Göttlich et al. (2014), Posner et al. (2014), and Vaghi et al. (2017), as well as the specifics of the experiment and patient population (Göttlich et al., 2014; Robbins et al., 2019). Despite differences, one common thread through the literature is the presence of a complex pattern of aberrant brain network communication in individuals with OCD. VC/VS DBS is believed to alter this pathological CSTC circuit function. For example, it alters cerebral glucose use in individuals with OCD (Rauch et al., 2006; Dougherty et al., 2016), and those alterations correlate with depressive (but not OCD) symptoms (Dougherty et al., 2016). Other groups have reported changes in cortico-striatal connectivity on functional MRI (Figee et al., 2013) or improvement in CSTC-related cognitive function after VC/VS DBS (Widge et al., 2019).

Thus, it may be possible to make VC/VS DBS more effective by identifying ways of more strongly disrupting targeted CSTC loops. Physiologically, this may mean disruption of abnormal oscillatory synchrony in the local field potential (LFP). LFP oscillations are argued to underlie many processes, including working memory, and even cognition in general (e.g., Miller et al., 2018). Oscillatory activity can be synchronous, or coherent, between brain regions, and this synchrony has been proposed to be a primary means by which regions in a circuit communicate (Fries, 2005, 2015). If this model holds and oscillatory synchrony is an index of communication between brain regions, then there may be CSTC hypersynchrony in individuals with OCD. High theta and beta subthalamic nucleus (STN) to cortical coherence has been reported in an individual with OCD (Wojtecki et al., 2017; using cortical MEG), but the synchrony theory has not been investigated within CSTC circuitry. In this way, establishing whether CSTC hypersynchrony exists in OCD may be a critical next step in understanding the disorder, improving treatments, and identifying useful biomarkers.

Similarly, disruption of oscillatory synchrony may be a mechanism of clinical DBS (Widge and Miller, 2019). For instance, in Parkinson’s disease, there is increased beta band activity in the STN, that beta power decreases with active DBS (Wingeier et al., 2006; Bronte-Stewart et al., 2009), and this decrease is in turn correlated with symptom improvement (Kühn et al., 2006, 2008; Ray et al., 2008). DBS in Parkinson’s specifically alters network-level LFP synchrony. For example, De Hemptinne et al. (2015) found a reduction in phase-amplitude coupling in the cortex with STN DBS in Parkinson’s patients, while Oswal et al. (2016) reported a reduction in cortico-STN coherence. In animal models, optogenetic neurostimulation increased oscillatory synchrony between brain regions, which was in turn causally linked to both changes in behavior and neurotransmission (Padilla-Coreano et al., 2019). To the degree that CSTC hyper-connectivity is reflected in hyper-synchrony, new stimulation methods to disrupt that synchrony may significantly improve the effectiveness of DBS (Widge and Miller, 2019).

Therefore, we proposed that delivering frequency mismatched stimulation to multiple areas within a CSTC circuit would disrupt OCD-related hypersynchrony/hyperconnectivity more effectively than single site simulation. Stimulation resets the phase of neural oscillations (e.g., Rosanova et al., 2018). Stimulating two regions at mismatched frequencies should thus disrupt synchrony, by preventing the phase of the oscillations in the two regions from aligning. The supplementary motor area (SMA) is a particularly promising second target for this mismatched stimulation. While traditionally associated with the motor CSTC loop (e.g., Nakano et al., 2000; Obeso et al., 2008), the SMA (and medial prefrontal cortex, more generally) also participates in decision-making linked to limbic/associative CSTC loops (Milad and Rauch, 2012; Dougherty et al., 2018). Further, transcranial magnetic stimulation (TMS) of the SMA is an effective treatment for individuals with severe OCD (Mantovani et al., 2010; Gomes et al., 2012; Carmi et al., 2018, 2019), implicating this area in the neuropathology of the disorder. Given the CSTC hyperconnectivity hypothesis, we hypothesized that OCD symptoms would be reflected in heightened coherence between these two regions. Further, we hypothesized that mismatched stimulation would break this hyper-coherence between VC/VS and SMA.

Here, we report the first patient in an early feasibility study^1^ combining VC/VS DBS with frequency mismatched stimulation of SMA in an effort to disrupt CSTC synchrony in treatment refractory OCD. The patient first received open-label VC/VS only stimulation, followed by a blinded phase of combined cortical and VC/VS stimulation, and finally an open-label combined stimulation phase. During the course of the study, daily LFP recordings from VC/VS and SMA were taken, allowing for the first known chronic recording of a cortico-striatal circuit in human. Using these recordings, we tested the hypersynchrony hypothesis, as well as the hypothesis that frequency mismatched stimulation could disrupt that hypersynchrony. These results are an important proof-of-principle toward understanding the mechanism of action for OCD neurostimulation, identifying biomarkers, and improving treatment.

### Patient History: Diagnoses, Symptoms, and Previous Treatment

The patient was a male in his 20s, who had previously received VC/VS DBS for treatment refractory OCD. Prior to the initial DBS surgery, the patient’s YBOCS was 29. He reported onset of OCD symptoms at approximately age 12–13, primarily of a mental ritualizing/obsessional type. Obsessions have included his symptoms themselves, counting, and symmetries. The patient had a past history of object-touching/rearranging compulsions, but at the time of his first course of DBS, he reported only mental rituals. Further, his OCD symptoms had sometimes been body-focused in ways that raised questions of body dysmorphic disorder (BDD).

Prior to beginning the first course of DBS, the patient was also diagnosed with treatment-resistant depression, with a baseline Montgomery-Åsberg Depression Rating Scale (MADRS) of 36. He reported substantial low mood and anhedonia, for the prior several years, combined with substantial anxiety. Symptoms also included difficulty concentrating when not using stimulants, low energy, some psychomotor slowing, and profound emotional numbing. The patient denied frank suicidality but had frequent thoughts/wishes of being dead. He had also previously carried the diagnosis of bipolar disorder, with past clinicians stating there were brief periods of hypomania. However, clinicians at the time of his first DBS surgery felt that symptoms previously labeled as hypomania were more correctly attributable to OCD/anxiety related racing thoughts. There were no identifiable distinct episodes of impulsivity, goal-directed activity, or decreased need for sleep.

The patient had been receiving weekly or biweekly cognitive behavioral therapy (CBT) and exposure with response prevention (ERP) for five years prior to the onset of his first course of DBS, conducted in a private practice. At the time of surgery, the patient continued that therapy with the same clinician, and was able to display numerous mindfulness and distress tolerance techniques.

The patient had also tried numerous serotonergic and dopaminergic medications: Paroxetine (four weeks), citalopram (2 weeks), mirtazapine (unknown time frame), fluoxetine (several weeks), lorazepam (unknown time frame), clomipramine (unknown time frame) were all trialed and discontinued due to intolerability of side effects. At the time of surgery the patient’s medications included: Fluvoxamine (400 mg), lithium (900 mg), amphetamine salt (20 mg), levomefolic acid (22.5 mg), lamotrigine (300 mg), olanzapine (27.5 mg) and levothyroxine (150 mcg). Additionally, the patient had undergone a course of rTMS for depression that he had not found helpful. At the time of his first DBS surgery, the patient had been undergoing bi-weekly maintenance electroconvulsive therapy (ECT) for depression, and had received around 50 sessions over the course of 2 years. The patient had found ECT to give him a slight mood lift. Despite these treatments, the patient continued to experience significant functional impairment, unable to attend community college, maintain significant employment, or live independently.

Roughly 3 years prior to enrollment in the present study the patient began his first course of bilateral VC/VS DBS treatment for OCD and depression. He showed initial improvement reaching his lowest YBOCS, 14, about 6 months after implantation. The patient’s MADRS dropped significantly as well, with his lowest score of 18 recorded over 2 years after surgery. However, this improvement was not sustained. Generally, his YBOCS was in the mid-to-high 20 s, and his MADRS in the high 20 s, to low 30 s. By the time of enrollment in the present study, his YBOCS and MADRS were back to baseline levels (27 and 37, respectively). Given that the patient was still experiencing significant functional impairment, a multidisciplinary review committee (Widge and Dougherty, 2015) felt that the patient met criteria for inclusion in the present study (full criteria are at https://clinicaltrials.gov/ct2/show/NCT03184454).

## Materials and Methods

All study procedures described below were reviewed and approved by the Institutional Review Board at Massachusetts General Hospital. The study was conducted under an Investigational Device Exemption from the US Food & Drug Administration (FDA).

### Surgery and Electrode Placement

The patient was implanted with bilateral electrodes targeting the VC/VS and SMA. VC/VS electrodes were implanted first, followed by SMA electrodes through the same burr hole. For VC/VS, the patient’s previously implanted leads (Medtronic model 3387 lead) were first removed, as we sought to use larger contacts to more efficiently activate capsular white matter and reach fibers running in the dorsal capsule. Using standard stereotactic surgery procedures, and coregistration of MRI and CT images, Medtronic model 3391 leads were implanted bilaterally targeting VC/VS. We sought to place contact 0 within the gray matter of the ventral striatum, 2 mm anterior to the posterior border of the anterior commissure. The lead trajectory was aligned with the internal capsule, so that contact 3 would be in the capsular white matter immediately adjacent to the caudate nucleus. Before securing the lead, we tested bipolar stimulation at up to 6 V (130 Hz, 150 μs pulse width) between all pairs of contacts, without adverse effects. There was no intraoperative hedonic or mirthful response.

**FIGURE 1:**
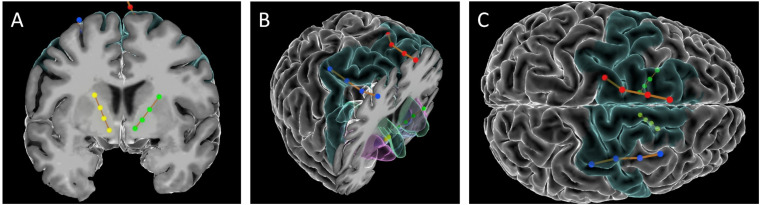
Images of the DBS and paddle leads rendered with the Multi-Modality Visualization Tool (Felsenstein and Peled, 2017; Felsenstein et al., 2019). Position of left (red) and right (blue) SMA leads, and left (green) and right (yellow) VC/VS leads. Brodmann Area 6 is colored in turquoise. **(A)** coronal slice showing position of VC/VS leads (subcortical regions not colored). **(B)** Angled view with cortical coronal slice. Caudate nucleus colored in green, nucleus accumbens (Nacc) in blue, and putamen in pink. **(C)** Superior view (left on top) showing cortical lead positions. Note: the right VC/VS lead is in the caudate nucleus and NAcc whereas the left VC/VS lead is more laterally placed in the putamen. No adverse effects of lead placement were observed with the patient.

Cortical paddles (Medtronic model 3986, Resume 4-contact paddle lead) were placed under direct visualization through the burr holes used to place the VC/VS electrodes. The surgeon (ZW) retracted the underlying cortex inferiorly and placed the paddles on the dorsal surface of the superior frontal gyrus (SFG). In this first patient, cortical lead placement was purely empirical, targeting the SFG just anterior to the motor strip, guided by the cortical landmarks visible to the surgeon. As with the deep leads, we performed test stimulations (50 and 130 Hz, 150 μs) at up to 4V through the cortical electrode to verify lack of adverse events. Before securing the electrodes, we recorded local field potentials (LFP) from both the deep and surface leads in each hemisphere through the intraoperative monitoring system (NeuroOmega, Alpha-Omega Systems, Nazareth, Israel; see Data Collection section below for more details). DBS and paddle electrodes were then secured using sutures on the dural edge, and burr holes were sealed with cranioplasty material. Final lead placement was confirmed by intraoperative x-ray, and post-operative CT scan. See Figure 1 for final lead locations.

In a subsequent surgery on the following day, two infraclavicular pulse generators (IPG; Medtronic Activa PC + S) were implanted bilaterally in the patient’s chest. The PC + S system was selected for it’s sensing/recording capabilities (discussed below). Given that the IPGs were only able to deliver pulses at one frequency to leads attached to the same device, the two VC/VS electrodes were attached to one device, and cortical electrodes were attached to the second device.

### Study Phases and Stimulation Parameters

**FIGURE 2:**
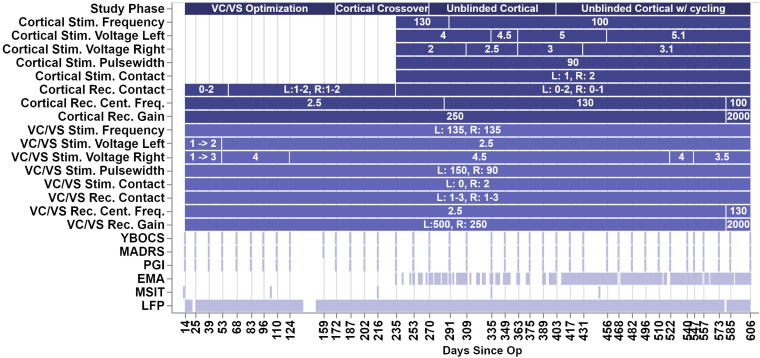
Figure depicts the timeline of study phases, changes in stimulation or recording parameters, and collection of clinical measures and LFP recordings. The *x*-axis values are days since the operation, and the ticks/labels denote days of clinical programming sessions. Note that there was no chronic cortical stimulation until day 235.

Over the course of almost 2 years, the patient progressed through several phases of a single-blind randomized cross-over study (see Figure 2 for timing of each study phase). In the VC/VS optimization phase (study days 0 to 172, as measured by the days since operation), he only received VC/VS stimulation. We identified the initial most effective contact and titrated VC/VS stimulation voltage according to the algorithm in Widge and Dougherty (2015). This also served as a baseline period in which cortical-striatal synchrony was measured in the absence of combined, mismatched stimulation. The original protocol called for a 2-week baseline phase after IPG implant, and preceding the VC/VS optimization phase, in which the pattern of LFP oscillations in the absence of stimulation could be established. However, on the day after the IPG was implanted, while recovering in the hospital, the patient reported depression and suicidality. He stated that this was due to withdrawal of his prior DBS therapy, that he was certain it would not be tolerable, and that he could not maintain his personal safety for any period of time. Consistent with the study protocol’s directives that suicidality was a reason to escape a patient from any given phase/procedure, his VC/VS leads were thus activated early. Thereafter, the patient declined to permit deactivation of VC/VS stimulation, even if only for a few moments, making stimulation-off recordings impossible to obtain.

In the blinded cortical crossover phase (days 172–270) the patient had biweekly clinical visits with the unblinded programmer (DDD). During one of these sessions (day 235), cortical stimulation was activated, unknown to the patient or to the researchers obtaining rating scales. At the onset of this phase (day 172), we performed an acute cortical optimization in which we identified cortical stimulation parameters. During the cortical optimization, the VC/VS electrodes remained on, using the same contact as previously programmed for clinical therapy, but set to a frequency of 135, 55, or 15 Hz. For each of those VC/VS frequencies, we programmed the ipsilateral cortical stimulation to a corresponding (slightly mismatched) frequency of 130, 50, or 10 Hz, respectively. The 5 Hz difference between frequencies was selected because this was the only spacing that could be achieved consistently at all three frequency bands given the limits of the pulse generator (at higher frequencies, only steps of 5 Hz were possible).

We then tested each contact of the cortical electrode, in a monopolar configuration, at 2, 4, and (if tolerated) 6 V. At each setting, the patient rated the change in his mood, anxiety, and overall energy level on a 1–10 scale. The settings producing the best clinical effect were retained, but the cortical electrodes/IPG remained inactive until the actual crossover at day 235. During the cortical optimization procedure, no evidence of seizure activity was detected by clinicians. This is unsurprising, as chronic epicortical stimulation has been trialed in psychiatric patients in multiple studies without epileptic complications (Kopell et al., 2011; Williams et al., 2016). In the open label, unblinded cortical phase (days 270–606) the blind was broken on the cortical stimulation while the patient continued to receive combined stimulation. The full course of the study ranged from day 0, or the day of surgery, to day 606, at which point the battery for the VC/VS IPG reached the cut-off for minimum battery life required to take recordings.

VC/VS stimulation parameters can be seen in Figure 2. The patient received constant unipolar stimulation at contact 0 (left hemisphere) and contact 2 (right hemisphere). VC/VS stimulation frequency was 135 Hz. VC/VS stimulation pulsewidth was 150 and 90 μs for left and right hemispheres, respectively. VC/VS stimulation voltage was gradually increased to 2.5 and 4.5 V for left and right hemispheres, respectively, during the VC/VS optimization phase.

Cortical stimulation parameters can be seen in Figure 2. From day 235 until day 403, the patient received constant unipolar stimulation at contact 1 (left hemisphere) and contact 2 (right hemisphere) of the cortical paddles. Cortical stimulation frequency was 130 Hz during the blinded cortical phase, until approximately 2 weeks after the patient was unblinded (from day 235 to 291). Due to patient complaints of what he described as “overstimulation,” the stimulation frequency was reduced to 100 Hz at day 291, where it remained for the remainder of the study. Complaints of “overstimulation” also resulted in the patient beginning day-night cycling of his cortical stimulation (turning it off at night) at day 403. See section “Clinical outcomes with deep brain and combined stimulation” for a more detailed description of the patient’s feelings of overstimulation. Cortical stimulation pulsewidth was 90 μs. Voltage ranged from 4 to 5.1 V for the left hemisphere, and from 2 to 3.1 V for the left hemisphere, limited in both cases by anxious distress at higher voltages.

Impedances were measured during clinical visits, and were within normal ranges. Mean (SD) impedance was 777.46 (18.54) Ω for the left VC/VS lead, 761.12 (42.89) Ω for right VC/VS, 757.64 (22.25) Ω for left cortical, and 1144.46 (79.22) Ω for the left cortical lead. There were no dramatic shifts in impedance throughout the study, and changes in impedance did not correspond to changes in power spectra.

### Data Collection

#### Clinical Outcome Measures

Clinical sessions occurred approximately every 2 weeks (see Figure 2). Stimulation settings were adjusted only during these sessions. The primary outcome variable was the YBOCS (Goodman et al., 1989). Key secondary outcomes were MADRS (Montgomery and Åsberg, 1979) and patient global impression of improvement (PGI-I; Yalcin and Bump, 2003). All were collected during the biweekly clinical sessions.

#### Ecological Momentary Assessment (EMA)

Midway through the present study, the patient enrolled in a separate study. The purpose of that study was to use data from the patient’s smartphone to obtain a more continuous measure of functioning than the sporadic clinical ratings. Among other measures, this study collected ecological momentary assessments (EMAs; Shiffman et al., 2008). Data collection for the EMA study began 151 days following surgery, with the first EMA collected 235 days following surgery. The EMA contained eight questions regarding the patient’s motivation and ability to perform tasks (e.g., “In the past 24 h; it was difficult for me to get anything done,” or “It was difficult for me to complete my morning routine”). These prompts were derived from the patient’s report of his primary symptoms. Questions were scored on a scale from 0 to 4, with 0 indicating the highest level of functioning (e.g., “extremely easy”) and 4 indicating the lowest level of functioning (e.g., “extremely difficult”). The scores for the eight questions were averaged to create a summary EMA score. The patient was prompted to take the EMA at least once a day but could choose not to participate.

#### Multi-Source Interference Task (MSIT)

During several clinical sessions (on days 13, 104, 216, 335, and 448, see Figure 2) the patient performed the Multi-Source Interference Task (MSIT; Bush et al., 2003; Bush and Shin, 2006). Considered to measure cognitive control, the MSIT produces robust subject-level behavioral and neural effects (Bush et al., 2003, preprint; Bush and Shin, 2006; González-Villar and Carrillo-de-la-Peña, 2017; Widge et al., 2019), which can be modulated through DBS of CSTC circuitry (Basu et al., preprint; Widge et al., 2019).

During an MSIT trial three numbers (between 0 and 3) were presented on the screen. Two of these numbers had the same value, and the other was different (e.g., 020 or 233). The patient’s task was to identify, via button press, the identity of the number that was unique, not its position. Trials were either congruent or incongruent. In congruent trials (e.g., 020), the unique number was in the same position as it’s corresponding keyboard position, and the other numbers were always ‘0’, which was never a valid response. In incongruent trials (e.g., 233) the unique number was in a different position than its corresponding position, and the non-unique numbers were always one of the other valid responses, such that incongruent trials contained multiple types of interference (position and response). Congruent and incongruent trials were presented together in a pseudo-randomized fashion, such that no more than two trials in a row ever shared the same condition or correct response finger. The patient performed 8 blocks of 48 trials each, for a total of 384 trials per run of the MSIT. The task was run using the Psychophysics Toolbox (Kleiner et al., 2007).

We analyzed MSIT response time (RT), as task accuracy is very high, and previous effects of DBS on MSIT performance have been shown in response time data. We grouped MSIT runs based on the stimulation phase/condition: VC/VS only stimulation with non-optimized settings (non-optimized VC/VS), VC/VS only stimulation after settings had been optimized (optimized VC/VS), and combined VC/VS and cortical stimulation (combined). Note that we only have MSIT runs during 100 Hz cortical stimulation. Trials were removed from analysis based on the criteria used in Widge et al. (2019). Namely, error and post-error (i.e., trials following an incorrect response) trials, as well as trials with RTs with a likelihood of less than 0.005 based on a fitted gamma distribution. We excluded 130 trials (0.07% of total trials), leaving 1790 trials in the analysis. Following Widge et al. (2019), we analyzed trial-wise RT in a generalized linear model (GLM) using a gamma distribution and identity link function, with conflict (congruent and incongruent) and stimulation condition (non-optimized VC/VS, optimized VC/VS, and combined) condition as the fixed effects. Collinearity between day since operation and stimulation condition was high. Further, adding day to the model containing conflict condition and stimulation condition (i.e., adding day to RT ∼ conflict condition + stimulation condition) did not add significant explanatory power (*F* = 2.32, *p* = 0.13), whereas doing the same for stimulation condition (i.e., adding stimulation condition to RT ∼ conflict condition + day since operation) did add significant explanatory power (*F* = 24.23, *p* < 0.0001). For these reasons we opted not to include day since operation in the reported model.

Conflict adaptation, or Gratton effect (Gratton et al., 1992), has been shown to be modulated by CSTC connected regions (Sheth et al., 2012). The patient failed to show the typical effect (slower RT when switching from a low conflict to a high conflict condition versus no switch), and therefore we did not examine changes in this effect across treatment.

#### Intraoperative Local Field Potential (LFP) Recordings

As stated previously, the high resolution intraoperative monitoring system (NeuroOmega, Alpha-Omega Systems, Nazareth, Israel) was used to record LFPs intraoperatively, after the electrodes had been implanted. We recorded simultaneously from all cortical and striatal contacts, with separate recordings for the left and right hemispheres. Two recordings per hemisphere were taken. Each recording was 2.25–2.5 min, with a sampling rate of 1000 Hz. LFP recordings were referenced against a needle electrode in the scalp.

#### Daily Local Field Potential (LFP) Recordings

Daily timer triggered recordings were taken by the cortical and VC/VS Activa PC + S devices throughout the course of the study. Recordings were taken every 6 h, yielding four recordings per day. Recordings were from a pair of contacts (bipolar montage) not used for stimulation. Recordings were 1 min long, at a sampling rate of 200 Hz (see Figure 2 for other recording parameters). Recordings were downloaded at least every 2 weeks. Note on Figure 2 that there are brief periods during the course of the study with missing LFP data, the largest of which occurred when the patient took an extended vacation. Given the potential for drift, the internal clocks of the cortical and VC/VS IPGs were re-synchronized with the programming device at each data download.

#### Saline Bath Testing and Artifact Subtraction

The recording/sensing capabilities of the Activa PC + S system have been utilized in preclinical (e.g., Connolly et al., 2015) and clinical (e.g., Swann et al., 2017; Huang et al., 2019; Veerakumar et al., 2019) studies. However, while the sensing capabilities in the PC + S system were designed to minimize the influence of stimulation artifacts, small artifacts remain (Stanslaski et al., 2012). Additionally, these original tests focused on measuring LFPs in the spectral domain; Stanslaski et al. (2012) state that results do not transfer easily to time domain, making phase-related analyses less reliable. For example, Swann et al. (2017) found broadband stimulation artifacts, as well as narrow band artifacts (with stimulation off) that were influenced by the sampling rate of recordings. Stimulation artifacts have caused some recent PC + S studies to analyze only stimulation-off recordings (e.g., Huang et al., 2019; Veerakumar et al., 2019).

Given that the recording and artifact removal capabilities of the PC + S device were not designed for our configuration (two IPGs delivering different frequency stimulation at the same time; Stanslaski et al., 2012), we specifically characterized the artifacts in our configuration in the absence of brain signal. We tested the recording and stimulation setting configurations used in the experiment in a saline preparation. We rejected the resulting artifact from the patient recordings (see LFP preprocessing and analysis below).

**FIGURE 3:**
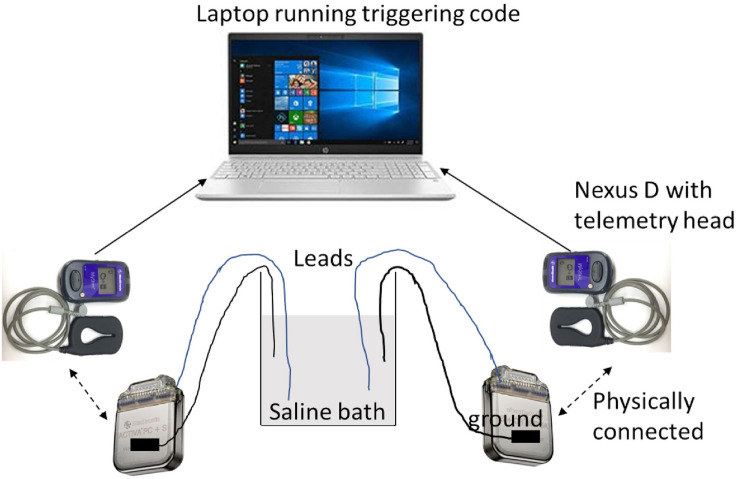
Schematic of saline bath preparation.

Saline testing used two Activa PC + S IPGs, with one electrode per IPG (Medtronic model 3387 and 3391). Settings were tested by taking simultaneous recordings, with the recording and stimulation settings used for the patient’s VC/VS leads in one IPG, and the settings used for the patient’s cortical leads in the other. Due to limited availability of leads, we were able to mimic only one hemisphere of the brain with a cortical and a VC/VS lead at one time. That is, this testing captured intra-hemispheric but not cross-hemispheric artifacts. Each lead had 4 contacts and were immersed in a saline bath. Each IPG was grounded through an alligator clip that was taped to the IPG body on one end via a metal foil and a resistor on the other end that was suspended in the same saline bath as the leads. Each IPG was connected via an antenna to a Nexus-D telemetry head which was in turn connected to a laptop (Figure 3). The recording settings on the IPGs were changed using a sensing programmer (SP) while the stimulation settings were changed using a clinician programmer (CP). Before starting any recordings, we first measured the impedances of both the electrodes with the CP. We ensured good contact on all leads, with impedance below 1000 Ω on at least 3 of the 4 contacts. We verified impedances again between recordings.

We manipulated frequency (cortical only), pulsewidth (150 μs for left VC/VS channel, 90 μs for other channels), configuration of the recording contacts (whether or not recording contacts directly flanked the stimulation contact), centering frequency, and gain (see Figure 2 for list of possible values). Sampling rate (200 Hz), and VC/VS channel frequency (135 Hz) did not vary over the course of the patient experiment, and thus were not varied in the saline test. To reduce the number of tests, only configurations used in the study were used in the saline test. We also took baseline (stimulation off) recordings, in which we assessed the change in the signal while varying only recording settings.

We took at least two recordings per setting configuration, on two separate days. Each recording was 2 min in length. We used custom written code in MATLAB to manually trigger recording in both the IPGs. Before sending the trigger command, we changed the recording settings (centering frequency, recording contact pair, gain) using the SP and the stimulation settings (stimulation current, frequency, pulse width, and lead contact) using the CP.

The saline bath recordings were preprocessed and power and synchrony were calculated using the same steps and criteria used for the patient recordings. See the LFP preprocessing and analysis section below for these criteria, and for a description of the methods used for artifact subtraction.

### Intraoperative LFP Preprocessing and Analysis

We calculated cortical-striatal synchrony for the left and right side, as a function of VC/VS depth. For the purposes of clarity/brevity and alignment with previous studies exploring cortical-striatal connectivity, we use the term cortical-striatal here, though at least one of the contacts is not technically in the striatal gray matter (but in VC/VS, more broadly). Data from the four contacts on each lead were first bipolar referenced, yielding 3 pairs. Intraoperative recordings were then epoched into one second segments, and bad epochs were identified and removed by visual inspection for artifacts. Epochs for the two recordings (from a given hemisphere) were concatenated.

We used the debiased weighted phase lag index (WPLI) as the measure of cortical-striatal synchrony. This measure was selected as a way to minimize stimulation artifacts in the daily LFP recordings from the device. Volume conduction of stimulation artifacts could create artificial synchrony between brain regions. We selected the debiased weighted phase lag index (WPLI) because it is less sensitive to this artifactual synchrony caused by volume conduction (Vinck et al., 2011). The WPLI, and other similar measures, use the principles proposed by the imaginary part of coherency (Nolte et al., 2004), which operates on the assumption that volume conduction has essentially no time lag (e.g., Stinstra and Peters, 1998), and therefore effects due to volume conduction will have zero phase lag. By using the imaginary components of the cross-spectral density, which are themselves phase shifted, phase synchrony with zero phase lag is removed (Nolte et al., 2004). The WPLI builds on the phase lag index (PLI; Stam et al., 2007), which is a measure of the asymmetry of the phase leads and lags between two signals, by weighting the contribution of phase asymmetries based on the magnitude of the imaginary component of the cross-spectral density (Vinck et al., 2011). Given that the WPLI can be positively biased (Vinck et al., 2011), the debiased estimator or squared WPLI was used.

To calculate WPLI each epoch was decomposed to its time-frequency representation (TFR) using Morlet wavelet convolution, with wavelet base frequencies from 5 to 50 Hz, in 32 logarithmically spaced steps, and the number of cycles characterizing a wavelet ranging from 3 to 7, in 32 logarithmically spaced steps. WPLI was calculated between the ipsilateral cortical and VC/VS leads.

### Daily LFP Preprocessing and Analysis

Local field potentials from the two recording contacts were bipolar re-referenced by the device internally prior to download from the IPG. All other LFP pre-processing and analysis was conducted using the MNE-Python suite (Gramfort et al., 2013).

Cortical and VC/VS recordings were temporally aligned using each IPG’s internal timestamp, which was reset during each data download session to reduce the amount of temporal drift. Given the reliance on the accuracy of the two devices’ timestamps, which do not have millisecond level precision, there is some uncertainty regarding the synchrony of the timing of the cortical and VC/VS signals. However, the temporal offset of the signals remains consistent within each pair of 1-min recordings. Phase synchrony is defined as a consistent phase difference, and thus can be calculated regardless of a constant shift/offset between two recordings. Only the portions of the recordings that overlapped temporally between the cortical and VC/VS IPGs were used in analysis. Therefore, while timer triggered recordings were each 1 min, the portion of the recording used in analysis was slightly less than 1 min in length. Recordings were band pass filtered between 5 and 50 Hz, in an effort to reduce the influence of stimulation artifacts. Additionally, given variations in the scale across recordings, each recording (within a channel) was normalized by scaling the band passed data to the interval from −1 to 1.

#### Spectral Analysis

Single recordings (5–50 Hz bandpass, normalized) were decomposed to their time-frequency representation (TFR) using Morlet wavelet convolution, and then averaged within the approximately 1-min recording to arrive at the power spectral density (PSD) for a given recording within each channel (VC/VS left, VC/VS right, cortical left, and cortical right). TFRs were calculated with wavelet base frequencies of 5–50 Hz, in 32 logarithmically spaced steps, and the number of cycles characterizing a wavelet ranging from 3 to 7, in 32 logarithmically spaced steps. These same TFR parameters were used for the synchrony analysis.

We then subtracted the artifact signal from the recording. This was done by subtracting the PSD from the saline bath recording matching the recording/stimulation settings used in the patient recording (PSD averaged across at least two saline recordings) from the PSD of a given channel for the corresponding 1-min patient recording.

Finally, for analysis of changes in power across the course of the study, we averaged the PSDs within each channel across the recordings for a single day to arrive at the average PSD for each day for the VC/VS left and right, and cortical left and right channels.

#### Cortical-Striatal Synchrony: Weighted Phase Lag Index

To calculate the WPLI the time-aligned, band-pass filtered, normalized recordings were epoched into one second segments. Within recording, each epoch was then decomposed to its TFR as above. WPLI was calculated between the ipsilateral cortical and striatal leads (separately for left and right hemispheres), then averaged across time to get the WPLI at each base frequency and hemisphere for a single minute recording. WPLI was calculated using the spectral connectivity function in the MNE python suite, with the wpli2_debiased option.

We then subtracted the artifactual WPLI from the left and right hemisphere WPLI of a given recording, by subtracting the WPLI across frequency from the saline bath recording matching the recording/stimulation settings used in the patient recording from the WPLI values for that 1-min patient recording.

To assess changes across the study, the WPLI from recordings on a single day were then averaged to get the average WPLI for that day for the left and right hemispheres.

#### Clinical Outcomes and WPLI Correlations

Based on our initial hypotheses, we explored the relationship between the clinical outcome measures (YBOCS, MADRS, PGI, EMA) and cortical-striatal synchrony (WPLI) in each frequency band. We calculated the mean WPLI (left and right hemispheres averaged) within each frequency band (theta, alpha, beta, gamma) for the nearest recording day that occurred prior to the day of the corresponding clinical outcome measurement. The day prior was used for clinical outcomes (YBOCS, MADRS, PGI), as on many occasions there were also stimulation settings changes that occured on the day the measure was taken (i.e., the recordings for that day reflected the stimulation settings, not the symptom burden over the prior week). In cases where the EMA did not occur on a clinical session day, the recordings from the day the EMA was taken were used to calculate WPLI. We then correlated the WPLI in each band to the clinical measures. Given multiple comparisons for each clinical outcome, a Bonferroni corrected *p*-value of 0.0125 was used to determine significance.

#### Random Forests Using LFP Features to Predict Clinical Outcomes

We conducted five-fold cross validated (using five equally sized groups) random forest regressions to predict each clinical outcome measure (YBOCS, MADRS, PGI, EMA) using features of the LFP recordings, as well as some recording and stimulation settings features. The number of dependent data points for each regression depended on the instances of the given outcome (total of 39 for YBOCS and MADRS, 38 for PGI, and 215 for EMA).

We created power and WPLI features from the LFP recordings. Power and WPLI for each recording, in each band (theta, alpha, beta, or gamma) were either averaged across a full day of recording (full day), or binned into the time of day they occurred (night: 12:00am to 6:00am; morning: 6:00am to 12:00pm; afternoon: 12:00pm to 6:00pm; evening 6:00pm to 12:00am). Within each of those groups, features were then created based on whether recordings contributing to the features were from the day prior (for clinical outcomes; day of for EMA only days) to the day a given outcome measure was taken, or were the average of the recordings across all the days in the 2 weeks prior the outcome measure. The total number of LFP features was 280. Features also included 12 recording and stimulation parameters that varied throughout the experiment (for a total of 292 features): cortical stimulation (on vs. off), cortical stimulation frequency, cortical and VC/VS (left and right separately) stimulation voltage, and left and right cortical recording channel names. Missing values were possible, as there was not always a recording that occurred during a given time of day when only a single day of data was used. Missing values were imputed using the mean from that feature.

We used the scikit-learn package in Python (Pedregosa et al., 2011) to perform the random forest analyses. The random forest was conducted using 2000 estimators, with 2 samples required to split the group (a low sample to split was chosen because of the low number of instances of the clinical outcomes). All possible features were used at each split. The model was calculated first with all the features, and then again using only the top 5 features (based on importance scoring) from the first model. We report model accuracy, *R*^2^, and feature importances for the model using the top five features only. Model accuracy is 100 minus the mean absolute percentage error (MAPE) in prediction of the outcome variable on the held-out test set. *R*^2^, or coefficient of determination, is essentially a measure of whether the model created using the training set is performing better than a constant model (i.e., using the training set mean) for predicting the values in the test set. *R*^2^, in this case, ranges from −1 to 1, with an *R*^2^ of 1 indicating perfect prediction, *R*^2^ > 0 indicating the model is performing better than the constant model, and an *R*^2^ < 0 indicating that the model is performing worse than the constant model at predicting the test set values. Feature importances are a normalized estimate of predictive power for each feature, based on the fraction of samples a feature contributes to, combined with decrease in error by splitting. Reported values for all measures are calculated as the average of the values for each of the five cross-validated test sets.

## Results

### Clinical Outcomes With Deep Brain and Combined Stimulation

**FIGURE 4:**
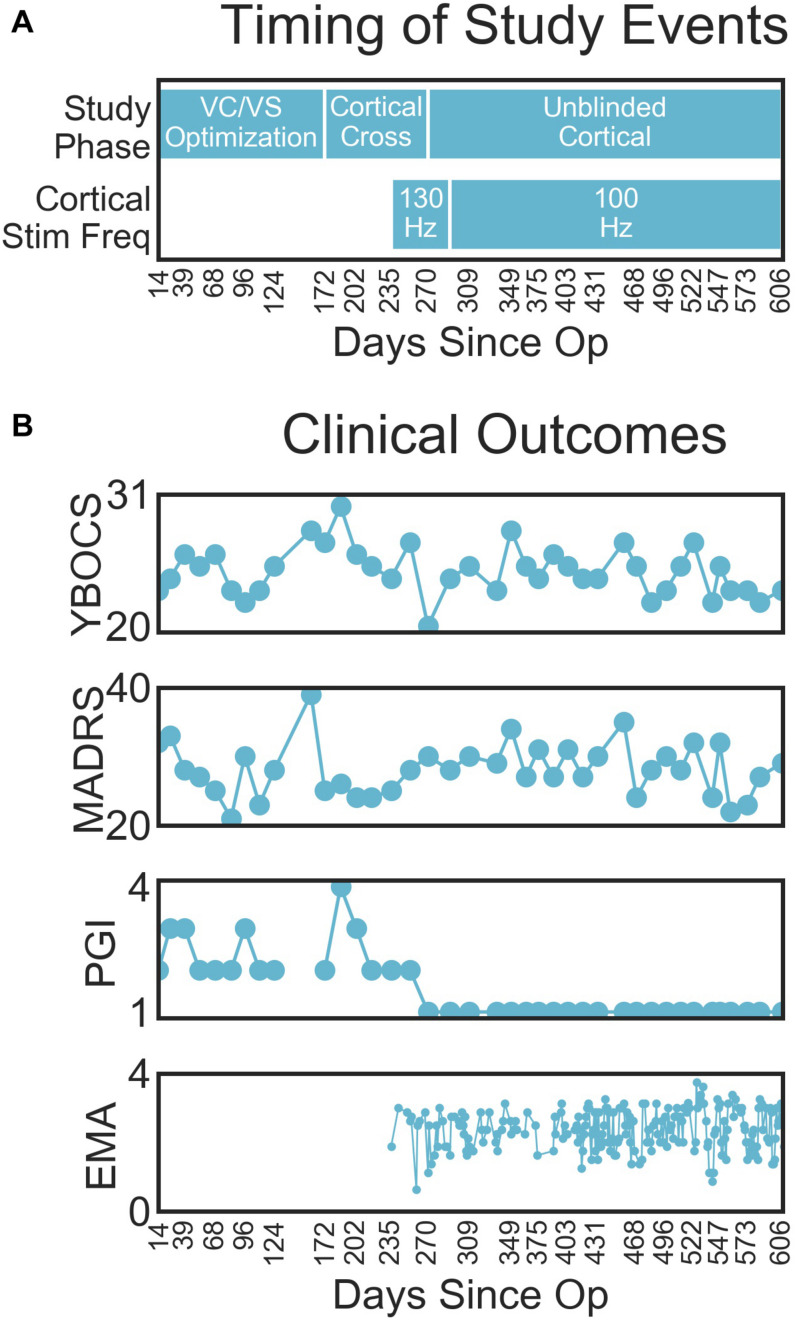
**(A)** Timing of important study events, for reference. **(B)** Clinical outcomes (from top to bottom YBOCS, MADRS, PGI, EMA) by days since operation.

Changes in clinical outcomes over the course of the study are displayed in Figure 4B (with the timing of study events, for reference in Figure 4A). The patient’s OCD symptoms changed modestly throughout the study (Figure 4B). The patient’s YBOCS was 27 at the pre-surgery baseline, at which point he was already receiving VC/VS DBS (he had a YBOCS of 29 prior to his first course of DBS). His mean YBOCS during the VC/VS optimization phase was 25.13 (*SD* = 2.13), dropping 13.34% from his score prior to any DBS. With the addition of cortical stimulation, his YBOCS dropped another point (*M* = 24.25, *SD* = 1.92), dropping a further 3% from his initial YBOCS of 29 (i.e., 16.38% change). YBOCS consistently fell below the criteria for severe OCD (YBOCS < 24) during his last four clinical sessions. There was no difference in mean YBOCS between the blinded and unblinded cortical phases. During acute cortical optimization the patient reported that with the addition of cortical stimulation he felt that he was more easily able to focus attention away from the OCD thoughts, and that it was as if the OCD was on the other side of a door or barrier, trying to get through, but that he was able to keep it behind the barrier. These subjective self-reports did not, however, translate to YBOCS improvement with chronic cortical stimulation.

The patient’s depressive symptoms appeared to improve with VC/VS stimulation, but did not improve further with the addition of cortical stimulation. By the end of his first course of VC/VS DBS, the patient continued to suffer from severe depression (MADRS > 34), with a MADRS of 37 at his pre-surgery baseline. During the VC/VS optimization phase, the MADRS dropped almost ten points (*M* = 27.33, *SD* = 4.61, 26.14% drop from baseline), with the patient no longer meeting criteria for severe depression. The MADRS rose slightly with the addition of cortical stimulation (*M* = 28.58, *SD* = 3.24, 22.76% drop from baseline).

The change in formal rating scales did not meet the standard criteria for YBOCS (35% drop) or MADRS (50% drop) response. Despite this, the patient felt that he was much improved with the addition of cortical stimulation. During the VC/VS optimization phase the patient’s PGI-I averaged somewhere between minimally and much improved (*M* = 2.47, *SD* = 0.64). With the addition of cortical stimulation, the patient consistently rated his symptoms as very much improved (PGI-I = 1).

At the time of this writing, the patient continues to live with family, does not maintain significant employment or volunteer activities, and has not returned to complete his education.

The patient did not experience any significant side-effects from either the VC/VS or cortical stimulation. He did report experiences of being “overstimulated” with the 130 Hz cortical stimulation. Due to these, his cortical stimulation was changed to 100 Hz at day 291, and he began cycling his cortical stimulation off at night beginning at day 403. The patient described this experience as an overfocused, anxious, or agitated state, with the patient also using terms like “racing thoughts” and “tunnel vision” to describe the feeling. The attending clinicians did not believe these represented a hypomanic state, given that they were not accompanied by impulsivity, euphoria, or pleasure-seeking. In theory, this “overstimulation” could be akin to the anxiety effects reported from VC/VS stimulation, except that VC/VS-related anxiety tends to have a very acute onset and the patient’s “overstimulation” feelings arose gradually. Moreover, even prior to this study, the patient’s obsessions often focused on his current mood state and his stimulation settings, i.e., the possibility that his settings were incorrect and that he might feel bad as a result. Thus, some of this might not reflect actual side effects, but his usual obsessional content. Indeed, the patient only began reporting feelings of overstimulation after being unblinded to the cortical stimulation, indicating that it may be more psychological than physiological.

### Multi-Source Interference Task (MSIT)

Both conflict condition (congruent and incongruent; *f* = 117.76, *p* < 0.0001) and stimulation condition (VC/VS non-optimized, VC/VS optimized, and combined; *f* = 1231.32, *p* < 0.0001) contributed significantly to the final model. RT was faster for congruent (*M* = 0.507 s, *SEM* = 0.004) than for incongruent (*M* = 0.691 s, *SEM* = 0.004) trials (β = 0.18, *z* = 34.36, *p* < 0.001), replicating the robust subject-level effects seen in the literature.

**FIGURE 5:**
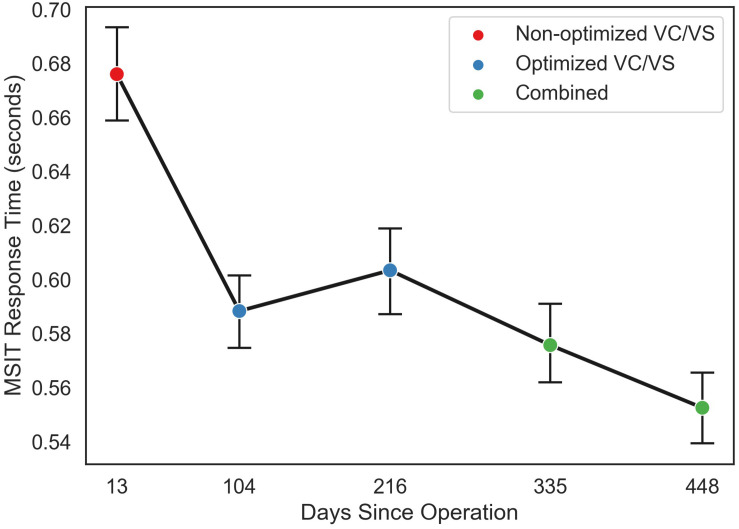
Mean response time (in seconds) for each MSIT run as a function of day since operation the patient performed the task, collapsed across congruent and incongruent trials. Color of points indicates the stimulation phase: VC/VS only prior to setting optimization, VC/VS only after optimization, and combined VC/VS and cortical (100 Hz) stimulation.

RT also differed as a function of stimulation condition (see Figure 5). RT was faster when the patient was receiving optimized VC/VS stimulation (*M* = 0.596 s, *SEM* = 0.005) compared to non-optimized VC/VS stimulation (*M* = 0.676 s, *SEM* = 0.009; β = −0.08, *z* = −10.33, *p* < 0.001). RT was fastest during combined stimulation (*M* = 0.564 s, *SEM* = 0.005), differing from both non-optimized (β = 0.11, *z* = 14.54, *p* < 0.001) and optimized (β = 0.03, *z* = 5.60, *p* < 0.001) VC/VS only stimulation.

### Daily LFP Recordings: Power and Cortical-Striatal Synchrony With Single-Site and Combined Stimulation

#### Intraoperative Cortical-Striatal Synchrony

**FIGURE 6:**
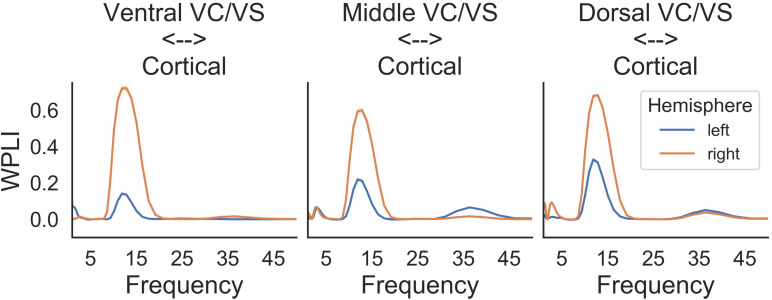
Intraoperative WPLI as a function of VC/VS depth.

A prominent alpha WPLI peak was detected intraoperatively using the high resolution OR rig (Figure 6). Alpha WPLI was more pronounced in the right hemisphere at each VC/VS depth. Right hemisphere alpha WPLI was relatively constant across VC/VS depth, whereas left hemisphere alpha WPLI was stronger more dorsally (near the head of the caudate). There was also a small gamma band WPLI peak, particularly in the left mid and dorsal striatum.

#### Saline Bath Test and Artifact Subtraction Results

**FIGURE 7:**
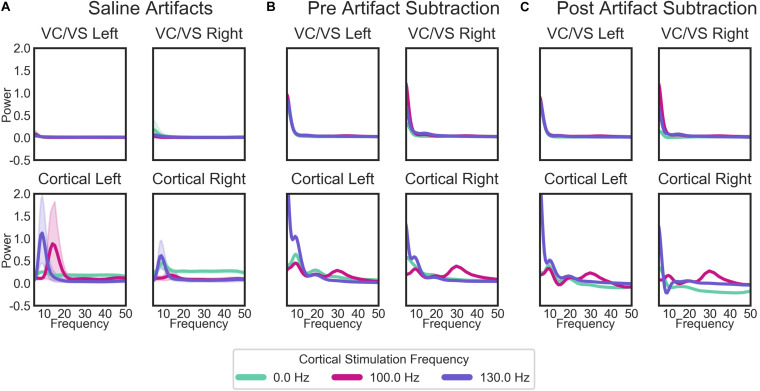
Average PSDs for: **(A)** the saline bath test (artifact) recordings; **(B)** the patient recordings prior to the removal of artifacts; and **(C)** the patient recordings after the subtraction of artifacts. For the patient recordings **(B,C)**, each plot represents the recordings from each of the patient’s four leads, labeled by the brain region (cortical or VC/VS) and hemisphere. Plots in **(A)** (saline recordings) are labeled by which region and hemisphere a given recording matched in terms of recording and stimulation settings. Lines in each plot are the average PSD for all recordings, separated by the cortical stimulation frequency. Cortical stimulation frequency of 0 Hz indicates that no cortical stimulation was on during that recording. Lines represent means for all saline bath **(A)** or patient **(B,C)** recordings in each group. Error bands represent 95% confidence intervals, calculated from 1000 bootstrapped samples.

When separated by the “cortical” stimulation frequency, there are noticeable artifacts in the “cortical,” but not “VC/VS,” saline recordings (Figure 7A). The spectrum of these artifacts differs depending on cortical stimulation frequency. For the patient recordings (Figures 7B,C), spectra also differ as a function of cortical stimulation frequency, particularly in the cortical recordings. These fluctuations largely remain after artifact subtraction, though the theta/beta peak appears to be much reduced. The 10 Hz peak in the cortical/right lead (Figure 7C, bottom right), at 130 Hz cortical stimulation is over-corrected, i.e., the saline artifact was larger than the same peak in the actual recording. Given this, 130 Hz cortical recordings showing a pronounced decrease in power in the 10 Hz range will not be interpreted as reflecting changes in brain signal.

**FIGURE 8:**
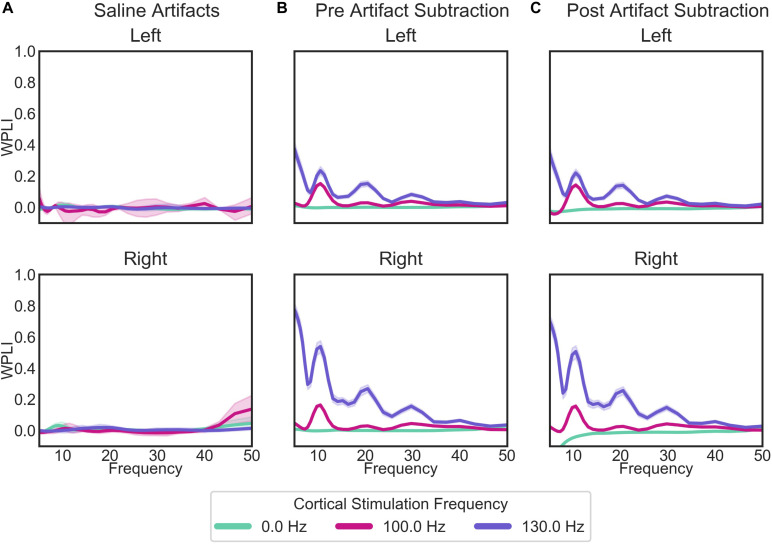
WPLI across frequency for: **(A)** the saline bath test (artifact) recordings; **(B)** the patient recordings prior to the removal of artifacts; and **(C)** the patient recordings after the subtraction of artifacts. Plots of patient recordings **(B,C)** indicate the cortical-striatal WPLI for the left and right hemispheres, with colored lines indicating the cortical stimulation frequency at the time of recording. Saline test plots **(A)** indicate whether the recording and stimulation settings for the IPGs matched those of the left or right hemisphere of patient recordings. Error bands represent 95% confidence intervals, calculated from 1000 bootstrapped samples.

While there are some marked artifacts in the power spectra, the synchrony spectra appear relatively artifact-free, as expected from a measure that is insensitive to volume conducted artifact (Figure 8A). In the patient recordings (Figure 8B), there are differences in WPLI with the type of cortical stimulation, which largely remain after artifact subtraction (Figure 8C). Contrary to our initial hypothesis, cortical-striatal synchrony increased with cortical stimulation, especially for 130 Hz stimulation. There is overcorrection in the lower frequencies of the right hemisphere, 0 Hz stimulation recordings (Figure 8C, top plot), therefore this will not be interpreted as hyposynchrony in the absence of cortical stimulation.

#### Daily LFP Spectral Analysis: Power Changes Over Time

**FIGURE 9:**
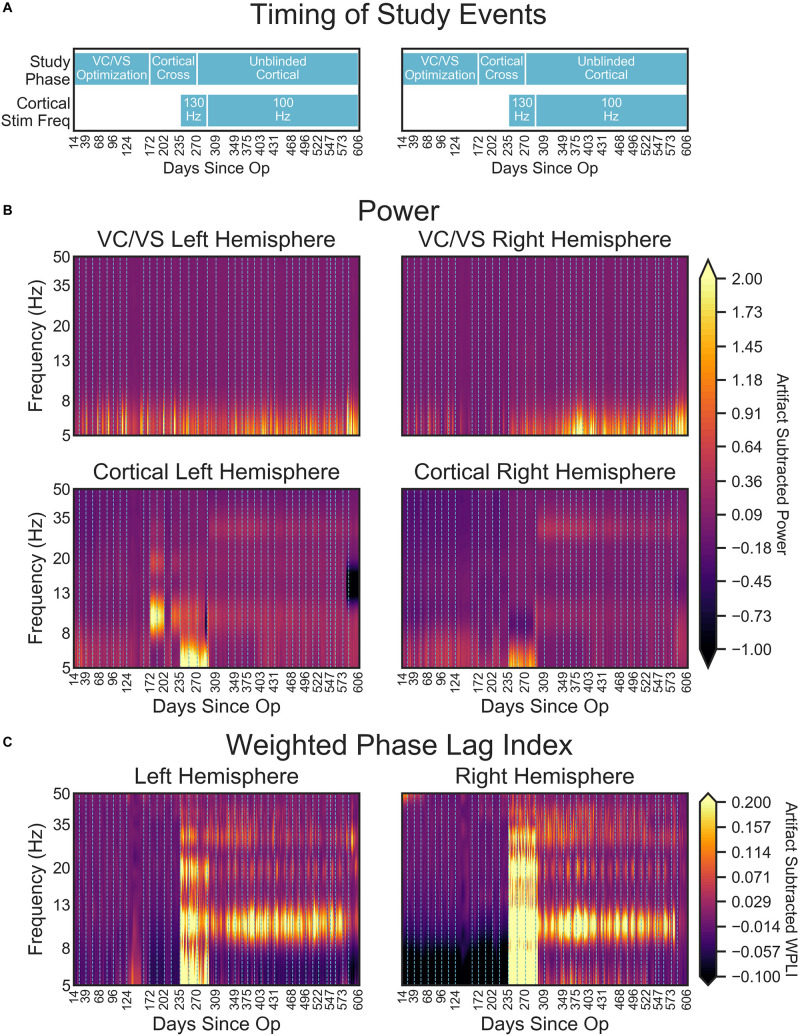
**(A)** Timing of important study events for reference. **(B)** Heatmaps denoting the artifact corrected power (5–50 Hz) across the course of the study. **(C)** Artifact corrected cortical-striatal synchrony (WPLI) across the frequencies tested, as a function of days since operation. To better show subtle changes in WPLI, the range used for the color map is –0.1 to 0.2. Dotted lines on heatmaps **(B,C)** indicate clinical sessions, during which stimulation and recording settings changed and clinical outcomes were taken (see Figure 2 above for timing of important settings changes). Areas with missing LFP recordings have been interpolated (e.g., between days 138 and 151).

VC/VS power spectra were relatively constant across stimulation settings, with a consistent peak in the theta range, and no other discernible peaks in the higher frequency bands (Figure 9B, upper panels, Figure 9A displays the timing of study phases for reference). Additionally, spectra were largely consistent across the left and right hemispheres.

Cortical power spectra, on the other hand, changed with study phases. Prior to turning on the cortical stimulation full-time, power for the cortical leads was relatively consistent across frequency and time (with some minor fluctuation in the theta band, Figure 9B lower panels). At day 235, when 130 Hz cortical stimulation was turned on, there was a dramatic shift in cortical power spectra. Interestingly, this shift resulted in increased power in the theta range, with an overall shift that looked much like the spectra of the DBS-on VC/VS recording. This change at approximately 5 Hz corresponds to the difference in frequencies between the cortical (130 Hz) and VC/VS (135 Hz) leads. The spectra shift again at day 291, when cortical stimulation is changed from 130 to 100 Hz. This shift results in the end of the increased theta power seen with 130 Hz stimulation, and some subtle banding in the alpha and beta/low gamma bands. These may correspond to the 35 Hz difference between the cortical and VC/VS stimulation.

There were also large shifts in cortical power spectra from day 172 to 202 and 216 to 235, which were more pronounced in the left hemisphere. No recording or stimulation parameter changes occurred at those times, other than the acute cortical optimization at day 172. Inspection of the non-normalized recordings indicated that the voltage values for those recordings were greatly increased relative to other recordings (by almost 100 fold). This may reflect a physical change in the contacts due to being stimulated for the first time, e.g., removal of accumulated protein deposits. However, the fact that these changes (including shifts in the scale of recordings) disappear between day 202 and 216, and then reappear between day 216 to 235, may indicate that there is also a neural component.

#### Daily LFP Cortical-Striatal Synchrony: Weighted Phase Lag Index

Consistent with our initial hypothesis, cortico-striatal synchrony changed more strongly than power across the study phases (Figure 9C). There were minimal differences between the left and right hemispheres. However, there does appear to be lower theta synchrony in the right hemisphere in the absence of cortical stimulation, which is not present in the left hemisphere. Given that this low theta synchrony only emerged after artifact subtraction (see Figure 8), and does not appear in the left hemisphere, its significance is uncertain.

Prior to the addition of cortical stimulation, WPLI was fairly equal across all frequency bands. This is in contrast to the intraoperative recordings (Figure 6), which showed WPLI peaks in the theta and alpha/low beta ranges. It is possible that this is a function of differences in the resolution of the recordings, but it may also indicate changes in synchrony when VC/VS stimulation is on (as is the case for the daily PC + S recordings) versus off (as is the case for the intraoperative recordings). Those changes would be consistent with our hypothesis that DBS disrupts cortico-striatal synchrony. The patient declined even temporary interruption of VC/VS stimulation, and thus we are unable to disentangle these possibilities at this time.

There is a dramatic increase in WPLI in the theta, alpha, beta, and low gamma bands when cortical stimulation is turned on full-time at 130 Hz (day 235). When stimulation is reduced to 100 Hz (day 291) this increase abates, although WPLI in the alpha, beta, and low gamma bands remains high relative to the other frequencies. Given the absence of WPLI artifacts in saline testing, these synchrony changes likely reflect true physiologic change. Contrary to our initial expectation, there was an increase in synchrony with combined VC/VS and cortical stimulation, and this increase was greatest when cortical stimulation was 130 Hz.

### Relationship of Power and Cortical-Striatal Synchrony to Clinical Outcomes

#### Clinical Outcomes and WPLI Correlations

**FIGURE 10:**
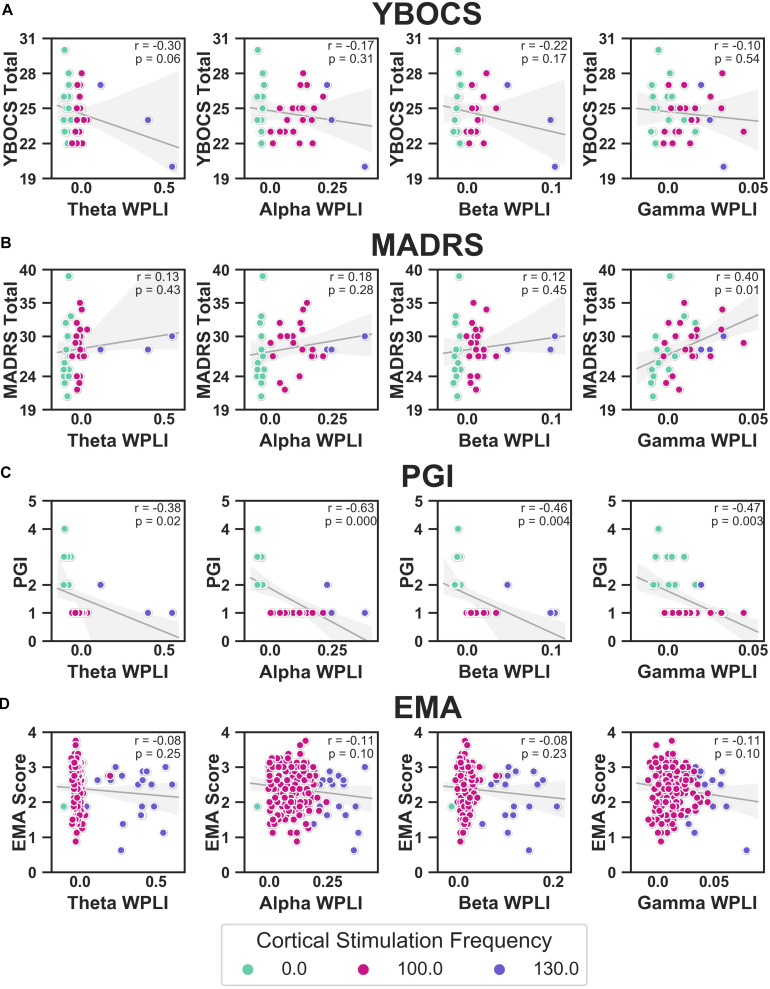
Correlations between YBOCS **(A)**, MADRS **(B)**, PGI **(C)**, and EMA **(D)** and WPLI in the theta (leftmost column), alpha (second from the left), beta (second from the right), and gamma (rightmost column) bands. Data points are colored by the cortical stimulation frequency. Linear regressions (gray lines) were fit to the full data set for that measure (i.e., not separated by cortical stimulation frequency), with the error bands indicating the 95% confidence interval. Pearson correlations were also calculated, and the corresponding *r* and *p*-values are displayed.

We correlated WPLI in each band (theta, alpha, beta, and gamma) to the clinical measures (Figure 10). Raw *p*-values are reported here; only *p*-values below the Bonferroni threshold of 0.0125 were considered significant. YBOCS improvement was correlated with higher WPLI in the theta band, but this did not reach significance (*r* = −0.30, *p* = 0.06) and may be driven by outliers. Improvement in MADRS was significantly correlated with lower WPLI in the gamma band (*r* = 0.40, *p* = 0.01). PGI correlations echoed the YBOCS, with improvement associated with increased WPLI in the alpha (*r* = −0.63, *p* < 0.001), beta (*r* = −0.46, *p* = 0.004), and gamma (*r* = −0.47, *p* = 0.003) bands. There were no significant correlations between EMA and WPLI in any band.

#### Random Forests Using LFP Features to Predict Clinical Outcomes

Table 1 contains the results of the random forest regressions predicting the clinical outcomes using LFP features. The models predicting MADRS (*R*^2^ = −0.14 ± 1.39) and EMA score (*R*^2^ = −0.01 ± 0.40) failed to perform better than a constant model. While average *R*^2^ for the model predicting YBOCS was positive, the 2 SD confidence interval included 0. We therefore concluded that the model did not meet performance criteria (*R*^2^ = 0.23 ± 0.28). This was likely due to a lack of variability in YBOCS scores across the course of the study; the mean predicted YBOCS scores with 94% accuracy, calculated as the average across the 5 cross validated test sets. In line with the correlation results, the model predicting PGI did perform better than the constant model (*R*^2^ = 0.77 ± 0.37), and was able to predict PGI of the test sets with 92% accuracy. Cortical-striatal synchrony in the gamma and theta bands appeared as important features in the model.

**TABLE 1:** Baseline accuracy indicates prediction accuracy for a constant model (i.e., using the mean to predict values), averaged across the 5 test sets.

Measure	Baseline accuracy	Model accuracy	Model *R*^2^	Top feature properties				Top feature importance’s
				Measure	Band	Recording time of day	Recording time period	
YBOCS	93.75%	94.25%	0.23 (±0.28)	SR Power	Gamma	Full Day	14 days prior	0.2870
				CL Power	Theta	Night	14 days prior	0.1945
				Left WPLI	Theta	Morning	1 day prior	0.1798
				SL Power	Theta	Evening	1 day prior	0.1723
				SL Power	Gamma	Evening	1 day prior	0.1665
MADRS	89.26%	89.06%	−0.14 (±1.39)	CL Power	Theta	Afternoon	14 days prior	0.2276
				Avg WPLI	Gamma	Evening	1 day prior	0.2165
				Right WPLI	Gamma	Full Day	1 day prior	0.2157
				Right WPLI	Gamma	Morning	14 days prior	0.2047
				Right WPLI	Gamma	Morning	1 day prior	0.1354
PGI	54.96%	91.80%	0.77 (±0.37)	Avg WPLI	Theta	Full Day	14 days prior	0.2360
				CL Power	Gamma	Afternoon	14 days prior	0.2250
				CR Power	Gamma	Evening	14 days prior	0.2051
				Left WPLI	Gamma	Morning	14 days prior	0.1922
				Right WPLI	Theta	Morning	1 day prior	0.1417
EMA	76.10%	77.48%	−0.01 (±0.40)	CL Power	Alpha	Evening	Day of	0.2240
				SR Power	Beta	Full day	Day of	0.2083
				CL Power	Alpha	Afternoon	14 days prior	0.1992
				Left WPLI	Gamma	Night	Day of	0.1849
				Avg WPLI	Theta	Afternoon	Day of	0.1836

*Model accuracy indicates the accuracy of the model using only the top five features. Model *R*^2^ (±2 SD), is the average test set coefficient of determination of the final model. *R*^2^ values > 0 indicate that the model is performing better than a constant model, and values < 0 indicate the model is performing worse. The top five features (from an initial model using all possible features) are broken apart here into their component parts. The measure column indicates which LFP measure the feature contains (SL power, striatal left power; SR power, striatal right power; CL power, cortical left power; CR power, cortical right power; left WPLI, right WPLI, or average of left and right WPLI); Band indicates the frequency band of the feature; Recording time of day indicates which time of day the recordings contributing to the feature were from (morning, afternoon, evening, night, or the full day); Recording time period indicates how many days of recordings preceding the day the clinical measure was taken were used in the analysis (14 days preceding, one day prior to outcome measure day- YBOCS, MADRS, PGI, or day of the outcome measure day - EMA only). Top feature importances indicates the feature importance (normalized estimate of the predictive power of each feature) of each of the top features, averaged across the 5-fold cross-validated models.*

## Discussion

We examined targeted CSTC network disruption with combined cortical (SMA) and VC/VS neurostimulation in one patient with treatment refractory OCD in a blinded crossover study. Chronic recording of the cortical-striatal circuit for almost 2 years allowed us to test the hypothesis that frequency mismatched stimulation would disrupt CSTC hypersynchrony, leading to a greater improvement in symptoms with combined stimulation compared to VC/VS stimulation alone. The patient is the first known case of chronic SMA stimulation, and of chronic combined cortical and VC/VS stimulation. The patient experienced no significant side-effects or adverse events with the addition of cortical stimulation. While this will need to be confirmed in future patients, these findings are a first step in establishing the safety of a combined cortical-subcortical approach to neurostimulation.

The patient experienced positive effects with acute combined cortical-striatal stimulation. Specifically, he reported an increase in the ability to divert focus away from OCD thoughts, which he did not feel with VC/VS-only stimulation. Throughout the study, the patient described cortical stimulation as the “icing on the cake” to traditional DBS. These positive effects did not translate into improvement in clinical outcomes with chronic combined stimulation, however. The patient did not respond to his initial course of standard DBS at the VC/VS target. Cortical and combined stimulation did not rescue this non-response. The patient’s YBOCS dropped 13% from baseline with striatal stimulation, and 16% from baseline with the addition of cortical stimulation. The patient’s MADRS dropped 26% with VC/VS stimulation, and 23% from baseline with the addition of cortical stimulation. While a single case cannot define a therapy’s potential, the lack of response to chronic combined stimulation was surprising, given the positive acute effects.

Despite the lack of significant movement in formal rating scales, the patient felt as though his symptoms had greatly improved with the addition of combined stimulation, as measured by the PGI. However, it should be noted that the shift to “very much improved” occurred in the session where the patient was unblinded to the cortical stimulation. In this way, the most parsimonious explanation of subjective improvement with cortical stimulation is a placebo effect. Additionally, despite subjective feelings of improvement, at the time of this writing the patient continued to show significant impairment in functioning, as well as moderate OCD and depressive symptoms. Nevertheless, the patient’s feelings of improvement may be important, given that they represent a change from previous treatments, and that the PGI-I has been shown to be related to more objective measures of symptom improvement in larger samples (e.g., Yalcin and Bump, 2003). Overall satisfaction with DBS therapy, despite a lack of response to the treatment has been described before (e.g., Denys et al., 2020). It is possible that this effect represents changes in overall mood, or the limitations of the YBOCS in terms of sensitivity to change at extremes of pathology (van Westen et al., 2020). It is also possible, however, that this is simply a subjective sense of, “I had brain surgery, so it must be doing something.” Regardless, overall satisfaction with the treatment even in the absence of response may serve a protective function, as it may represent a decrease in hopelessness, which is correlated with long-term adverse outcomes such as suicide (Papakostas et al., 2005; Beck et al., 2006).

The patient’s cognitive control, as measured by performance on the MSIT, also appeared to improve with combined stimulation. In line with previous research showing improved performance with VC/VS DBS (Basu et al., preprint; Widge et al., 2019), the patient’s response speed improved when he was receiving optimized VC/VS stimulation. With the addition of cortical stimulation he showed an additional quickening of response time, compared to optimized VC/VS stimulation alone. It should be noted, though, that the patient did appear to show an effect of time, such that he improved as day since operation increased (see Figure 5). Our study design meant that stimulation condition and day since operation were highly collinear. Therefore, we are unable to dissociate the improvement seen with stimulation condition from an improvement with time. However, Widge et al. (2019) found no differences in RT between multiple MSIT runs conducted an average of 88 min apart. It is unlikely that such effects would emerge at much longer time delays, such as those seen in our study. Therefore, differences between VC/VS only stimulation and combined stimulation may reflect an additional boost to cognitive control with the addition of cortical stimulation. This finding tracks with our finding of subjective symptom improvement with combined stimulation, and the possibility that the YBOCS may not have been sensitive enough to detect changes in our patient’s OCD pathology. Namely, there may have been subtle shifts in some of the cognitive deficits thought to underly OCD (e.g., Robbins et al., 2012; Shin et al., 2014; Voon et al., 2015; Vaghi et al., 2017) which resulted in the subjective improvement felt by the patient, but which were too subtle to produce significant changes in traditional rating scales. This finding also tracks with prior studies implicating medial prefrontal cortex in the cognitive deficits seen in OCD (Haber, 2003; Cocchi et al., 2012; Robbins et al., 2012; Vaghi et al., 2017; Robbins et al., 2019).

This study represents the first chronic recording of the cortical-striatal circuit in a human. Using these recordings we were able to measure cortical-striatal synchrony continuously for nearly 2 years. In line with our initial prediction, frequency mismatched stimulation did in fact alter cortical-striatal synchrony. However, this alteration was in the opposite direction of our initial prediction - frequency mismatched stimulation actually increased cortical-striatal synchrony. Further, the increase in synchrony was greater when the two frequencies were closer together (130 and 135 Hz), versus when they were farther apart (100 and 135 Hz). While there were power changes with acute cortical stimulation, synchrony changes only emerged with chronic stimulation. These findings remained even after the removal of stimulation artifacts. Additionally, the increase in cortical-striatal synchrony (in the alpha, beta, and gamma bands) was associated with an increase in the patient’s subjective feelings of improvement, with LFP features (especially synchrony) predicting PGI with 92% accuracy in a random forest regression. Synchrony was not significantly related to either OCD or MDD symptoms. Importantly, given the n of 1 and the absence of baseline and stimulation off recordings, any conclusions regarding changes in synchrony are extremely tentative. Future research is needed to elucidate the influence of combined (and arguably, single-site) stimulation on the synchrony of neural oscillations, and its relationship to symptom improvement.

If the finding holds, one possibility for the unexpected increase in synchrony with combined stimulation is that neural elements may have imprecise or broadly tuned frequency responses, or responses that become insensitive to mismatch at high driving currents (Fröhlich, 2015). At the relatively high stimulation intensities used in clinical DBS, a small mismatch between driving frequencies may essentially be zero mismatch from the biological system’s perspective. Another possibility is that the DBS frequency may entrain the endogenous frequency, as was observed for narrowband gamma in Swann et al. (2016). Finally, rather than causing disruption (by forcing two oscillators out of phase), it may be that the separation between frequencies can actually entrain activity at the difference between the two driving frequencies, an effect that some have proposed could be exploited therapeutically (Grossman et al., 2017).

Subjective symptom improvement with increases in cortico-striatal synchrony was not in line with our initial hypothesis that OCD arises from CSTC hypersynchrony. While this finding clearly requires replication, one explanation is that the hyperconnectivity hypothesis represents an oversimplified view of the neurobiology of OCD. As discussed previously, there is evidence of both hyper and hypoconnectivity (Gürsel et al., 2018), which may be partially a function of which CSTC loop (e.g., motor, associative, or limbic loops: Obeso et al., 2008; Krack et al., 2010; Milad and Rauch, 2012; Lapidus et al., 2013) the regions showing aberrant connectivity are in Harrison et al. (2009); Göttlich et al. (2014), Posner et al. (2014), and Vaghi et al. (2017). In this way, it is possible that our patient’s specific pattern between VC/VS and SMA was one of hypoconnectivity, and combined stimulation did move his networks toward a more normal/healthy connectivity pattern.

Establishing an individual’s specific pattern of connectivity, therefore, may be a critical step in developing personalized treatments for OCD. However, establishing this pattern does no good if there is no means of restoring the communication to “normal” levels. Neurostimulation, and in particular combined stimulation, offers a unique means of directly influencing connectivity between regions. Despite the direction, our results suggest that the communication between CSTC regions, as measured by phase synchrony, may be altered by neurostimulation. Further, our results indicate that these alterations can potentially be sustained across long periods of time, while the patient is receiving stimulation. Thus, this case supports the possibility of using DBS to deliver personalized, network-level therapy.

### Limitations, Lessons Learned, and Future Considerations

The results of the present study are limited in that it is difficult to assess whether changes in power and synchrony are a result of actual changes in brain signal, or are artifacts of stimulation. This is in part a result of the imperfect artifact rejection from the device recordings (Stanslaski et al., 2012; Swann et al., 2017), amplified in this case by the fact that some of our optimal stimulation contacts did not permit the use of the preferred “flanking dipole” recording configuration. Further, while we attempted to subtract the artifact signal from our recordings using saline bath test recordings, these methods were imperfect. Most notably, we did not have enough leads to test the full four lead configuration in saline, potentially missing artifacts that only emerge with that full configuration. Despite imperfections, we do feel that the process of establishing the signal in the absence of brain signal (which to our knowledge has not been reported before in the DBS literature) may be an important check when making claims about the effect of DBS on neural oscillations in the presence of potential stimulation artifacts. Further, effective artifact subtraction/removal will almost certainly be critical for developing closed-loop therapies, which are a critical next step in advancing neurostimulation (Bilge et al., 2018; Widge et al., 2018).

To the prior point, we believe this study is the first to report an attempt at multi-structure chronic recording through two implanted PC + S systems. One of our unpleasant surprises was that this implant configuration could not be combined with real-time data streaming. The two implanted neurostimulators exhibited cross-talk during streaming attempts, whether using the base PC + S system (Sensing Programmer) or the more advanced Nexus-D toolkit. Starting streaming sessions from a second IPG immediately ended the streaming from whichever IPG we had contacted first. It is unclear whether this will continue to be a limitation in future generations of sensing systems, which may benefit from continued advances in medical implant communication infrastructure. There is a move toward wireless programming even for clinical applications, which necessitates the development of devices that can flexibly switch bands to prevent cross talk.

One solution to the problem of stimulation artifacts is simply taking recordings with stimulation off. This is the approach that has been taken in previous studies (Huang et al., 2019; Veerakumar et al., 2019), and will be our approach moving forward. However, taking stimulation off recordings presents potential challenges/drawbacks. The first is the potential unblinding of the participant during a randomized protocol. There is also the potential that the changes in synchrony due to combined stimulation only occur while stimulation is on. One indication that this may be the case comes from studies showing that the beneficial effects of VC/VS DBS for OCD are not sustained when stimulation is turned off in a blinded fashion (Luyten et al., 2016), and from our own studies showing rapid cognitive change from DBS discontinuation (Widge et al., 2019). Therefore, we also plan to take recordings with stimulation on. While this brings us back to the issue of stimulation artifacts, we believe that having corresponding stimulation on and off recordings would only be beneficial.

The data from this specific patient are limited in that there were no baseline recordings taken prior to turning on VC/VS stimulation. Baseline recordings would have helped establish the level of cortical-striatal synchrony in our patient in the absence of an intervention. The original protocol called for 2-weeks of baseline recordings. However, as discussed previously, while recovering in the hospital the patient reported suicidality, which he attributed to cessation from his prior DBS therapy. From then on, the patient declined any deactivation of VC/VS stimulation, and as such, we were unable to obtain any recordings in the absence of stimulation. This is also the reason we were unable to obtain stimulation off recordings throughout the course of the study. Psychiatric DBS patients generally tolerate turning the device off [e.g., 14 out of 14 participants in Widge et al. (2019) tolerated having their stimulation turned off], so DBS on/off comparisons will likely be possible in the future, and will clarify the baseline, non-stimulation recording characteristics.

The results are also potentially limited in the specific patient selected as the first participant. Given that the patient did not respond to his initial course of VC/VS DBS, the approving physicians felt that there was hope of improvement with the addition of combined stimulation. In hindsight, the lack of response to prior VC/VS DBS may instead have been an indication that the patient would also be more likely to be a non-responder to other types of neurostimulation. Further, the presentation of the patient’s OCD symptoms is particularly challenging, in that his compulsions are largely mental and thus difficult to target for exposure. It is very hard to distinguish some of these compulsions from ruminative preoccupation. This pattern may have made it less likely that the patient would respond to treatment, and is an important caution for DBS patient selection generally.

Moving forward, we also plan on implementing EMA style assessments of OCD and other symptoms, which are an important way of capturing more frequent variability in symptoms [see Walz et al. (2014) for a review of their use in anxiety disorders]. We will also attempt take corresponding LFP recordings in an effort to more successfully model changes in symptoms using the features of the LFP. The EMA used in the present study was limited in that it did not specifically measure OC symptoms, but instead was an assessment of motivation and the ability to perform daily tasks. Further, the EMA only began being collected mid-way through the study, meaning that important baseline levels were not established. For the next patient, we plan to collect a wide range of baseline questionnaires and EMAs prior to initiating treatment. Theoretically, it should also be possible to then titrate future EMAs to just the areas in which the patient shows the most impairment, making the EMAs more user friendly. As previously discussed, there are significant nuances and (likely) individual differences in the complex pattern of aberrant connectivity in individuals with OCD. Therefore, establishing an individual’s specific pattern of connectivity may help improve neurostimulation therapies for psychiatric disorders through targeting brain regions which show dysfunctional connectivity. While there are almost certainly others, we see two methods of implementing this type of targeting. The first is the use of diffusion tensor imaging (DTI) and tractography. This type of MRI was not possible in the first patient due to his chronic indwelling hardware, but we anticipate collecting it in future patients. Using the patient’s tractography between certain CSTC seed regions, we may be able to more specifically target both sub-cortical and cortical electrodes. The second is the through measuring cortical-striatal synchrony intraoperatively, in real-time. Electrodes (especially cortical) could be placed in areas showing the most pronounced synchrony patterns (either high or low synchrony, depending on the patient).

## Conclusion

Psychiatric disorders, including OCD, are network disorders. We have shown that those networks can potentially be safely manipulated with multi-site continuous stimulation, and measured over periods of years with currently available technologies. Although the patient was not relieved of his psychiatric symptoms to the extent expected, our results are important safety and feasibility evidence toward a more network-oriented and personalized approach to DBS.

## Data Availability Statement

Preprocessed data and analysis script are available at: https://github.com/tne-lab/Olsen-et-al-2020. Raw data and preprocessing scripts can be made available upon request.

## Ethics Statement

The studies involving human participants were reviewed and approved by Institutional Review Board of Massachusetts General Hospital. The patients/participants provided their written informed consent to participate in this study. Written informed consent was obtained from the individual(s) for the publication of any potentially identifiable images or data included in this article.

## Author Contributions

AW conceived, developed, and supervised all aspects of the study, with significant input from DD. SO wrote the manuscript with support from AW and IB and performed the final data analysis with significant input from MTB, IB, and AW. AW, DD, CC, and TD performed the clinical care aspects of the study. ZW performed the study surgical procedures. MTB, IB, AK, MJB, AR, AG, and EH carried out the study procedures and collected the data. IB performed the saline bath test study. MTB conceptualized and created the data analysis procedures. IB, AK, MJB, AR, AG, and EH provided the data analysis support. NP and AR performed the MRI analysis and developed the MMVT software. JB conceived of and carried out the separate study of which the ecological momentary assessments are reported here. ME, IS, KF-H, and JS performed the data collection and some analysis of the ecological momentary assessment study. All the authors contributed to the article and approved the submitted version.

## Conflict of Interest

AW, DD, and TD are named inventors on patent applications related to deep brain stimulation and oscillations, including at least one application related to the subject of this paper. AW and DD have received consulting income and/or research support from Medtronic, which manufactured the devices used in this study. Medtronic had no financial or technical involvement with this specific research. TD discloses honoraria, consultation fees, and/or royalties from the MGH Psychiatry Academy, BrainCells Inc., Clintara, LLC., Systems Research and Applications Corporation, Boston University, the Catalan Agency for Health Technology Assessment and Research, the National Association of Social Workers Massachusetts, the Massachusetts Medical Society, Tufts University, NIDA, NIMH, and Oxford University Press. None of those entities manufactures technology or products used in the study. The remaining authors declare that the research was conducted in the absence of any commercial or financial relationships that could be construed as a potential conflict of interest. The reviewer SS declared a past co-authorship with several of the authors DD, and AW to the handling editor.

## Footnotes

1 https://clinicaltrials.gov/ct2/show/NCT03184454
